# Resistance to Thyroid Hormone in a Boy with a Severe, Complex, Congenital Heart Defect (CHD) Requiring Multiple Cardiac Surgeries—Whether and How to Prepare Child for the Surgery

**DOI:** 10.3390/jcm14041209

**Published:** 2025-02-12

**Authors:** Anna Fedorczak, Beata Kruk, Anna Mazurek-Kula, Łukasz Kępczyński, Renata Stawerska

**Affiliations:** 1 Department of Endocrinology and Metabolic Diseases, Polish Mother’s Memorial Hospital–Research Institute, 93-338 Lodz, Poland; anna.fedorczak@outlook.com; 2 Department of Cardiology, Polish Mother’s Memorial Hospital–Research Institute, 93-338 Lodz, Poland; b.slap@interia.pl (B.K.); qla@op.pl (A.M.-K.); 3 Department of Genetics, Polish Mother’s Memorial Hospital–Research Institute, 93-338 Lodz, Poland; lukasz.kepczynski@iczmp.edu.pl; 4 Department of Pediatric Endocrinology, Medical University of Lodz, 93-339 Lodz, Poland

**Keywords:** resistance to thyroid hormones, thyroid hormone receptor β, cardiac defect, child, surgery

## Abstract

**Background**: Resistance to thyroid hormones (RTH) is a rare, genetically determined disease characterised by reduced tissue sensitivity to thyroid hormones (THs). It is caused by mutations in genes encoding the receptors for thyroid hormones, α (THRα) or β (THRβ), the distribution of which varies between tissues. Therefore, patients present with elevated TH levels with unsuppressed TSH levels, and symptoms of both hypothyroidism and hyperthyroidism may be present. **Methods**: Hence, we report the case of a boy with a complex, cyanotic, congenital heart defect who was also diagnosed with TH resistance syndrome. **Results**: Because of the clinical features of hyperthyroidism in preparation for cardiac surgery, thiamazole was administered, resulting in the normalisation of TH effects on the α-receptor for HTs. Due to the effectiveness of the proposed treatment, it was further introduced before the further stages of cardiac surgeries. **Conclusions**: The management of RTH is a constant challenge for clinicians and must be individualised.

## 1. Introduction

Thyroid hormone (TH) resistance syndrome (RTH) is a rare, genetically determined disease characterised by reduced tissue sensitivity to THs [1]. The condition results from mutations that impair the function of thyroid hormone receptors (THR), leading to dysregulated hormone action in various tissues. The estimated incidence of the disease is one in 40,000 live births [2,3]. The most commonly identified cause of RTH is point mutations in the *THRB* gene, which encodes the thyroid hormone receptor beta (THRβ), while mutations in the *THRA* gene, encoding thyroid hormone receptor alpha (THRα), are much less frequent [4]. Clinically, RTH presents with a biochemical profile characterised by elevated levels of circulating thyroid hormones (fT3 and fT4) with unsuppressed thyroid-stimulating hormone (TSH) levels. Due to the differential distribution and function of TH receptors in various tissues, affected individuals may exhibit symptoms of both hypothyroidism (e.g., short stature) and/or hyperthyroidism (e.g., tachycardia—heart rate depends on the effect of THs on the non-defective THRα) [5,6]. In most cases, symptoms are sparse, and patients do not need treatment, whereas patients exhibiting symptoms may require treatment with β-blockers, antithyroid medications, or synthetic thyroid hormones.

However, to our knowledge, no case has yet been described of a patient with resistance to thyroid hormones and a serious congenital heart defect. Furthermore, the optimal perioperative management of patients with RTH undergoing surgical interventions remains undefined. Thus, the aim of this study was to present a currently 5-year-old boy with a complex, congenital heart defect requiring multistage cardiac surgery, who was also diagnosed with RTH due to mutations in the gene encoding THRβ.

## 2. Materials and Methods

The data were collected from a retrospective analysis of the medical, laboratory, and genetic records of a patient who was under the care of the Department of Cardiology, the Department of Cardiac Surgery, and the Department of Endocrinology and Metabolic Diseases Clinics at the Polish Mother’s Memorial Hospital–Research Institute in Lodz. The study was carried out with prior informed parental consent. As this was a retrospective study, the Bioethical Committee of the Medical University of Lodz declared that special ethical approval was not required.

Serum TSH, FT4, and FT3 concentrations were measured by the electroimmuno-chemiluminescent method (ECLIA), Roche, Elecsys ^®^ Systems 1010/2010/modular analytics E170. For TSH, the analytical sensitivity was 0.005 μIU/mL, ranging up to 100 μIU/mL, with an intra-assay coefficient of variance (CV) of 1.5–8.6% and accuracy of 1.1–3.0%. The analytical range for FT4 was 0.023–7.77 ng/mL, the intra-assay CV was 1.4–2.9%, and the accuracy was 2.7–6.6%. For FT3, the analytical range was 0.26–32.55 pg, the intra-assay CV was 3.7–9.5%, and the accuracy was 3.8–11.2%.

Sanger sequencing of THRB exons 3, 4, 5, 6, and 8 was performed on patients’ genomic DNA isolated from the whole blood sample. The primers were designed via Primer3 input software (version 0.4.0). PCR amplification was conducted via PCR Master Mix (Promega Corporation, Cat. No. M7423, Madison, WI, USA). Following the amplification step, the resulting products were purified before sequencing. The BigDye Terminator v3.1 Cycle Sequencing Kit (Thermo Fisher Scientific, No. 4337455, Waltham, MA, USA) was used for sequencing, which was conducted on a 3500 Genetic Analyzer instrument (Thermo Fisher Scientific, Waltham, MA, USA). The heterozygous, pathogenic variant NM_000461.5(THRB):c.947G>A p.(Arg316His) was detected. Variant classification was performed using ACMG-AMP guidelines with ClinGen modifications.

## 3. Detailed Case Description

A 12-month-old boy with a complex, cyanotic, congenital heart defect was admitted to the Department of Cardiology at PMMH-RI in order to prepare for the subsequent stage of surgical treatment—Glen bidirectional surgery. The boy’s complex heart defect was characterised by a double outlet right ventricle (DORV) with a ventricular septal defect (VSD), pulmonary stenosis (PS), and hypoplasia of the left ventricle (LV).

Birth history: The baby was born from a first pregnancy and first birth at 38 gestational weeks by caesarean section, with a weight of 3240 g. He was prenatally diagnosed with a heart defect (DORV + VSD + PS). At birth, the baby presented features of circulatory insufficiency and an infusion of prostaglandins was administered; then, captopril and spironolactone were introduced to the treatment with clinical improvement. In the neonatal period, the child had poor weight gain and temporarily required feeding by gastric tube. At 4 months of age, the surgical removal of an atrial septal defect (ASD) and right ventricular outflow tract (RVOT) dilatation was performed.

Upon admission, the patient was in good general condition. His height was 75 cm (25th–50th percentile) and his weight was 8.5 kg (10th–25th percentile). Arterial blood pressure was 95/60 mmHg and blood saturation was 85–88%. Physical examination revealed tachycardia, with a heart rate of 125 bpm; no other abnormalities were noted. The following tests were performed before qualifying him for the surgery:

Electrocardiographic examination (ECG): Sinus rhythm, regular, 130/min. PQ = 160 ms, QRS = 60 ms, QTc = 413 ms. Features of right ventricular hypertrophy with overload.

Chest radiograph: Bilateral small interstitial thickening dependent on congestive changes. Otherwise, lung areas were without focal changes. Diaphragm and diaphragmatic–rib angles were free. The cardiac silhouette was enlarged in the transverse dimension with a cardiothoracic ratio (CRT) of 0.69. The left mediastinal outline was convex—most probably a thymus outline.

Echocardiography: Mitral valve with a narrow mitral annulus with a limited opening to approx. 5 mm with a flow of up to 1.7 m/s; tricuspid valve with normal flow and moderate central regurgitation and at the base of the STL; in IAS, a cavity with a diameter of approx. 12 mm with a left-to-right leak without restriction was found. In IVS, a cavity with a diameter of approx. 9 mm for the restoration of septo-aortic continuity of approx. 14 mm was found. We observed an aortic valve with laminar flow; a hypoplastic pulmonary trunk with predominant subvalvular stenosis up to 4.8 mm and significant flow acceleration up to 5.3 m/s; and pulmonary branches at the level of bifurcation of about 5 mm each. The thymus was visible in the subcostal view. IVC—8 mm; SVC—7 mm; Ao asc—11 mm; Ao thoracic—6.6 m; Ao abdominal—6 mm; aortic arch left; Ao desc without features of stenosis. Heart cavities: right ventricle above normal, left ventricle at the lower limit of normal; in 2D RVDd—28 mm, LVDd—16 mm RV/LV—1.75; contractility was good; E = 73%. Conclusions: double outlet right ventricle (DORV) + ventricular septal defect (VSD) + pulmonary artery (PA) hypoplasia + pulmonary stenosis (PS) + mitral valve (MV) hypoplasia + left ventricle (LV) hypoplasia; status post-surgical removal of an atrial septal defect (ASD) and the right ventricular outflow tract (RVOT) dilatation.

Blood test results revealed elevated levels of free thyroid hormones FT3 (6.39 pg/mL; N: 2.41–5.5) and FT4 (2.3 ng/mL; N: 0.96–1.77) with the absence of thyrotropin suppression (TSH = 2.93 mIU/L; N: 0.7–5.97) (Table 1).

Therefore, an endocrinology consultation was requested and the diagnostics were expanded to antithyroid antibodies and thyroid ultrasonography. Anti-peroxidase (anti-TPO, 20.87; N: <34 IU/mL), anti-thyroglobulin (anti-TG, 11.74, N: <115 IU/mL), and anti-TSH receptor (anti-TSHR, <0.3; N: <1.75 IU/L) antibodies were negative.

Thyroid ultrasound: The thyroid gland is typically located on the neck; the lower poles of both lobes do not descend below the sternal notch. The left lobe measured 23 × 8 × 8 mm and the right lobe measured 19 × 9 × 6 mm. The thickness of the isthmus was 2 mm. The echogenicity of both lobes was slightly hypoechoic and homogeneous. No focal changes were visualised. The organ showed a normal vascularisation pattern.

Thyroid hormone resistance (RTH) was suspected and genetic testing was ordered. Sanger sequencing of exons 3, 4, 5, 6, 8 of the *THRB* gene revealed heterogenous NH_000461.4: c.947G>A, p.(Arg316His), a variant classified as pathogenic, which confirmed the diagnosis of RTH. As a mutation indicating RTH was diagnosed and a pituitary RMI would have required general anaesthesia; we waived the pituitary RMI until the boy is older.

To reduce the potential risk of cardiac arrhythmias during surgery in a boy with a low cardiac ejection fraction and tachycardia, in preparation for cardiac surgery (BG Glenn type), we decided to use thiamazole to decrease FT4 and FT3 levels and the effect of their excessive concentration on THRα receptors. We initially administered the drug at typically used doses (0.5 mg/kg body mass) for 7 days, without achieving the normalisation of FT3 and FT4. Therefore, a dose of 2 mg/kg body mass thiamazole was applied for a period of 7 days, achieving the normalisation of FT3 (4.71 pg/mL) and FT4 (1.65 ng/mL) as well as normal heart function. At the same time, excessive TSH secretion was observed (12.12 mIU/L) (Table 1). Elevated TSH levels did not constitute an endocrinological contraindication to the procedure (the dysregulation between TSH and FT3 and FT4 was determined by disease). Thus, the patient was qualified for Glenn surgery; the course of the procedure was successful and no arrhythmias or other cardiac complications were observed. In the postoperative period, the treatment (thiamazole) was gradually reduced and the test results returned to baseline.

Given the clinical efficacy of the proposed management, the same treatment (thiamazole in dose 2 mg/kg body weight for 10–14 days) was implemented prior to subsequent stages of cardiac surgeries (Table 2). In the periods between surgeries, the patient did not receive any endocrine treatment. We did not consider therapy with propranolol, because propranolol reduces the cardiac ejection fraction and is not recommended in patients with a univentricular heart. At 5 years of age, the staged treatment was completed.

Currently, the boy remains under cardiological and endocrinological care. He displays features of hyperactivity, with normal intellectual development. His growth rate and height are within the normal range with respect to age and sex (107 cm, 50th–75th percentile). In the patient’s family, the same point mutation was detected in the boy’s father, with no known impact on the cardiovascular system or other aspects of health.

## 4. Discussion

Thyroid hormones (THs) act through α (THRα) and β (THRβ) receptors. Their distribution is variable and depends on the type of tissue: the α type predominates in the brain, the β type in the liver, and both types are present in the myocardium [1,7]. The metabolic effects of TH mainly depend on TRβ1, whereas the cardiac effects of TH are mediated by TRα [8]. TH resistance (RTH) may be caused by a mutation in *THRβ*, or, far less frequently, in *THRα* [8]. Patients with this syndrome usually have elevated FT4 and FT3 concentrations with normal or increased TSH levels [9,10]. The described clinical features of RTH include goitre, tachycardia, emotional disturbances, attention deficit hyperactivity disorder, hyperkinetic behaviour, hearing loss, low body mass index, short stature, and delayed bone age, among others [9]. Tachycardia occurs in 75–94% of patients with RTH and is often the finding that prompts diagnosis [11]. A few cases of the coexistence of TH and atrial fibrillation have also been reported [12,13]. Sometimes the aetiology of hyperthyroidism may be difficult to establish—like in a newborn with RTH, born to a mother with Graves’ disease [14]. Distinguishing between RTH and TSH-producing pituitary tumours may be challenging, as there are no significant differences in TSH and TH concentrations in both conditions. Therefore, pituitary imaging as well as the results of TRH testing and genetic testing would be helpful [15].

There is no therapy that corrects a defect in thyroid hormone receptor function. Patients with adequate compensation by enhanced TH production without thyrotoxic symptoms do not require treatment [16], whereas patients with hyperthyroid and/or hypothyroid symptoms may require ß-blockers, antithyroid drugs, or synthetic forms of thyroid hormones. The rationale for the antithyroid drugs treatment in patients with RTH remains controversial. The administration of antithyroid drugs further raises TSH and may consequently induce goitre and or pituitary thyrotrophic hyperplasia [17]. On the other hand, several cases of successful thiamazole therapy in children diagnosed with RTH and hyperthyroidism symptoms were also reported. One of them described that therapy with antithyroid drugs in a 2-year-old patient diagnosed with RTHβ improved behaviour, sleep, and body weight and reduced tachycardia [16]. Another case report presented a girl treated with thiamazole followed by levothyroxine replacement therapy, achieving notable improvement in intelligence, verbal skills, and behaviour [18]. The introduction of thiamazole and propranolol in a 15-year-old girl with hyperactivity, behaviour disorders, and tachycardia improved clinical symptoms but also increased TSH levels [19]. In conclusion, indications for treatment in RTH patients should be discussed individually.

Ultimately, we did not find any publications on how to prepare a patient with RTH for surgery. As our patient was diagnosed with the c.947G>A, p.(Arg316His) variant in one allele of the *THRβ* gene, the symptoms presented by the boy were attributed to an excessive effect of THs on the THRα, which was not affected by the mutation. Given the fact that the boy presented selective symptoms of hyperthyroidism (tachycardia), we decided to introduce cardiac surgery preparation with thiamazole. This approach aimed to reduce the impact of THs on the THRα, which mainly mediates the cardiac effects of THs [8].

It is well known that thyroid dysfunction is associated with elevated cardiovascular risk [20]. The cardiovascular effects of TH excess include an increase in heart rate, stroke volume, and cardiac output [21]. Hyperthyroidism enhances the risk of atrial fibrillation, cardiovascular disease, and heart failure [22]. Therefore, the aim of preparing patients with thyroid dysfunction for surgery is to normalise TH levels before surgical intervention whenever possible [23,24]. Being concerned about the impact of THs on THRα in the heart, we decided to proceed with this case similarly to patients with hyperthyroidism, where antithyroid drugs are recommended as a fundamental treatment and preoperative approach [24]. Antithyroid drugs act to suppress the synthesis of thyroid hormones by inhibiting the enzyme thyroperoxidase, which adds iodine to tyrosine residues on the hormone precursor—thyroglobulin. Thiamazole, as well as propylthiouracil, are absorbed immediately and accumulate in the thyroid gland, inhibiting the synthesis process of THs [25]. Recently, only thiamazole has been recommended as an antithyroid drug due to the elevated risk of hepatitis induced by propylthiouracil [25].

To date, no similar case report has been reported in the literature. We conclude that this description is important to provide some clues for the management of patients with RTH requiring complex surgical interventions.

## 5. Conclusions

The majority of patients with RTH do not require treatment and the observed abnormalities in hormonal test results do not constitute a contraindication to surgery. However, the simultaneous occurrence of a severe complex heart defect and RTH in a child presenting with selective clinical symptoms of hyperthyroidism prompted us to apply atypical preoperative treatment (antithyroid drugs), which resulted in the normalisation of TH effects on the THRα. The management of RTH is an ongoing challenge for clinicians and must be individualised.

## Figures and Tables

**Table 1:** Results of the patient’s hormonal panel.

Hormone	Range	Diagnosis	Surgery Preparation (Thiamazole 0.5 mg/kg)	Surgery Preparation (Thiamazole 2 mg/kg)	Between the Surgeries
TSH [mIU/L]	0.7–5.97	2.56	4.87	12.12	2.55
FT3 [pg/mL]	2.41–5.5	7.16	6.41	4.71	6.72
FT4 [ng/mL]	0.96–1.77	2.4	2.3	1.65	2.55

TSH—Thyroid-stimulating hormone; FT3—Free Triiodothyronine; FT4—Free Thyroxine.

**Table 2:** Stages of treatment for a complex, congenital heart defect.

No.	Name of Performed Surgery	Age
1.	Surgical removal of an atrial septal defect (ASD) and right ventricular outflow tract (RVOT) dilatation	4 months
2.	Glenn bidirectional surgery	13 months
3.	Cardiac catheterisation with the implantation of a Valeo stent into proximal segments of pulmonary arteries	20 months
4.	Cardiac catheterisation with closure of the pulmonary artery trunk using an Amlatzer Vascular Plug	2 years
5.	Fontan operation without fenestration—anastomosis of the inferior vena cava with the right branch of the pulmonary artery using a vascular prosthesis under extracorporeal circulation	4 years
6.	Cardiac catheterisation with closure of a veno-venous fistula in the left venous angle with an Amlatzer Vascular Plug	5 years

## Data Availability

Data are contained within the article.
